# A network model for biofilm development in *Escherichia coli K-12*

**DOI:** 10.1186/1742-4682-8-34

**Published:** 2011-09-22

**Authors:** Andrew A Shalá, Silvia Restrepo, Andrés F González Barrios

**Affiliations:** 1 Grupo de Diseño de Productos y Procesos (GDPP), Departamento de Ingeniería Química, Universidad de los Andes. Carrera 1E No. 19ª14 Bogotá, Colombia; 2 Laboratorio de Micología y Fitopatología (LAMFU), Facultad de Ciencias Biológicas, Universidad de los Andes, Universidad de los Andes. Carrera 1E No. 19ª14 Bogotá, Colombia

## Abstract

**Background:**

In nature, bacteria often exist as biofilms. Biofilms are communities of microorganisms attached to a surface. It is clear that biofilm-grown cells harbor properties remarkably distinct from planktonic cells. Biofilms frequently complicate treatments of infections by protecting bacteria from the immune system, decreasing antibiotic efficacy and dispersing planktonic cells to distant body sites. In this work, we employed enhanced Boolean algebra to model biofilm formation.

**Results:**

The network obtained describes biofilm formation successfully, assuming - in accordance with the literature - that when the negative regulators (RscCD and EnvZ/OmpR) are off, the positive regulator (FlhDC) is on. The network was modeled under three different conditions through time with satisfactory outcomes. Each cluster was constructed using the K-means/medians Clustering Support algorithm on the basis of published Affymetrix microarray gene expression data from biofilm-forming bacteria and the planktonic state over four time points for *Escherichia coli K-12*.

**Conclusions:**

The different phenotypes obtained demonstrate that the network model of biofilm formation can simulate the formation or repression of biofilm efficiently in *E. coli *K-12.

## Background

In natural, medical or engineering environments, bacteria often exist as sessile communities called biofilms [1], which are exquisite structures caused by a genetically programmed developmental process in which each stage entails dramatic modifications at the genetic, biochemical, and phenotypic levels [2]. This phenotype enables bacteria to adhere and anchor to surfaces in aqueous environments [3], so the cells acquire specific advantages when invading tissues, such as antiobiotic and shear stress resistance. It is estimated that biofilms are involved in 65% of human bacterial infections [4], since cells in biofilms are 1000 times more resistant than cells in the planktonic state, making medical treatments fail [1].

Many authors [5] have identified five steps in biofilm formation: (i) reversible attachment, (ii) irreversible attachment, (iii) maturation-1, (iv) maturation-2, and finally (v) dispersion [6]. Each step requires reprogramming of gene expression, and this reprogramming occurs in response to environmental changes [7,6]. The full development of the biofilm includes the existence of a three-dimensional structure made of a polysaccharide matrix that contains water channels for transporting nutrients and removing waste [5,7]. Different organelles play big roles during biofilm formation at different stages on the bacterial surface, e.g. during reversible attachment flagella propel cells toward the surface to overcome electrostatic interactions [6]. The irreversible attachment process requires the cells to lose their flagella and develop adhesive organelles such as curli or fimbria to attach to the surface [1]. Finally, the production of colanic acid capsules allows the three-dimensional structures of the mature biofilm to be constructed [8]. For specific organelles to appear at each step in the proper order, expression of these organelles must be coordinated and thus regulated by a subset of external signals, regulators and secondary messengers, e.g. flagellum biogenesis requires the positive regulator FlhD/FlhC (FlhDC), environmental conditions such as appropriate temperature, osmolarity, and pH, the presence of acetate and the transcription factor HdfR, etc. [6]. Many types of genes with different functions seem to be involved in biofilm formation [9].

Recently, there has been a dramatic upsurge of research on biofilms aimed at preventing or controlling their formation or eradicating them [10], since they are often deleterious and more complex to treat than planktonic forms owing to their high resistance to antibiotics [2]. It is therefore important to understand the genetic basis of biofilm formation in order to find effective ways to prevent it. Whole genome profiling for each stage provides invaluable information about the underpinnings of the regulation process [1,11,5,4]. Domka et al. [12] compared the gene expression in cells forming biofilms and suspended (planktonic) cells over time in *E. coli *K-12.

The gene expression profile could be further interpreted to elucidate the gene regulation network and variations in its topology over time. Once established, mathematical models can be used to predict the system dynamics and understand it deeply. These models are based on different approaches such as conservation mass balance and the mass action law, and involve the use of ordinary differential equations, or stochastic kinetics in cases where it is no longer possible to apply mass action assumptions. However, these techniques demand extensive development if a unique solution is required, and this kinetic background cannot easily be obtained [13]. For this reason, approaches that do not demand so much information such as Boolean based network kinetics constitute an ideal way of gaining a deeper understanding of the dynamics of gene networks, because this technique just requires the topology of the network for different time points [14-16].

The value of a Boolean logic network modeling resides in translating a continuous to a discrete dynamic system [13]. However, this discretization is only possible when each node response can be described by binary variables [14], which is the general case for gene transcription regardless of the biological system. Boolean networks allow regulatory networks to be modeled and analyzed efficiently, making strong simplifying assumptions about the structure and dynamics of a genetic regulatory system [17]. In this model each cluster (a group of genes) at a given time can display one or other of two states, on or off. The expression of a cluster A at time *t *+ 1 is modeled by a Boolean function, whose entries are the expression at time *t *of all *K *clusters related to cluster A. Generally, *K *≤ *N *where *N *is the total number of clusters obtained. After a succession of times, the system traces its history in a space of states [14].

Domka et al. [12] compared the differential gene expression over time when *E. coli *form biofilms, and gene expression between cells in suspension and biofilms. However, this study was limited to determining the proportion of genes induced and repressed between 2.5 and 5 fold, without identifying the most important networks or pathways for biofilm formation. Therefore, the aim of the present study is to build a network model of biofilm formation in *E. coli *K-12, to identify clusters and regulators during biofilm formation and to analyze the dynamics of each cluster to corroborate the results previously obtained through whole genome profiling.

## Results and discussion

Ten clusters for four time points were obtained with similarity levels greater than 82%. We then identified relevant clusters regarding biofilm formation for *E. coli *(Table 1) to calculate the weight matrix (Table 2). The connection between expression (model) and phenotype (biological conclusions) is based on the expression and presence of the positive regulators (clusters A and D).

**Table 1:** Main features of each cluster in biofilm formation

Cluster Name	Features of cluster
**A**	FlhDc regulator and all flagella, curli and capsule genes
**B**	EnvZ/OmpR regulator
**C**	RcsBC regulator
**D**	H-HS regulator, Fimbria gene (FimA), DnaAKJ and GrpE
**E**	cold shock protein, Transfer RNA
**F**	QseBC
**G**	Basic genes for surviving i. e. NADH dehidrogenase, & hdfR
**H**	Basic genes for surviving i. e. tryptophan genes, & LrhA
**I**	30-50S ribosomal subunits proteins
**J**	5S, 23S, 16S Operons

Some major genes and features for each cluster that takes part in biofilm formation. Each cluster was obtained from cluster analysis of the microarray data of the whole *E. coli *K-12 genome.

**Table 2:** Weight matrix for biofilm formation

Cluster number	A	B	C	D	E	F	G	H	I	J
**A**	1	-1	-1	1	0	1	-1	-1	0	0
**B**	0	1	1	0	0	0	0	0	0	0
**C**	0	1	1	0	0	0	0	0	0	0
**D**	1	-1	-1	0	0	0	0	0	0	0
**E**	0	0	0	0	0	0	0	0	1	1
**F**	1	0	0	0	0	0	0	0	0	0
**G**	0	0	1	0	0	0	0	0	1	1
**H**	0	0	1	0	0	0	0	0	1	1
**I**	1	1	1	1	1	1	1	1	0	1
**J**	1	1	1	1	1	1	1	1	1	0

Correlations between clusters obtained from different literature that used microarray data for *E. coli *K-12. 0, 1 and -1 represent neutral, positive and negative interactions, respectively.

**Figure 1:**
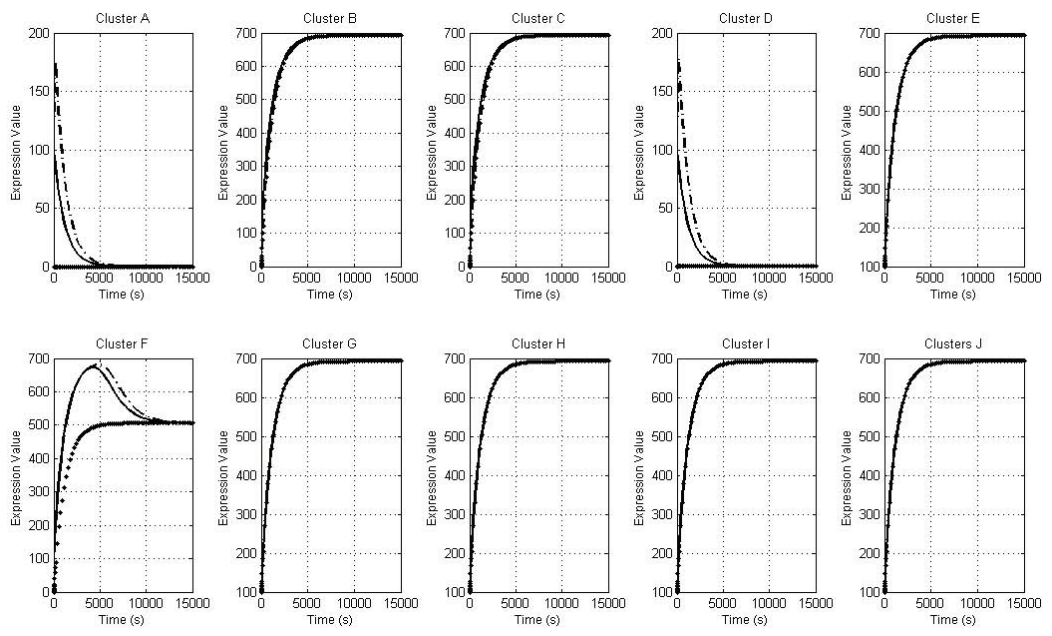
**Profiles for the ten clusters**. Plots for the ten clusters (A-J) show the expression profile (y-axis) vs. time (x-axis) for three scenarios: condition I (-), condition II (-.) and condition 3 (...). Biofilm is not formed under the null scenario because the biofilm positive regulators (clusters A and D) are repressed [expression value (ev) = 0] for four negative regulators (clusters B, C, G and H) [ev = 700]. Quorum sensing (cluster F) is at basal level [ev = 500].

Overall, we found no effect on the expression trend for the different initial conditions (Table 3) in Clusters E, G, H, I & J as they reached the active state, regardless of the initial population (Figure 1). This result corroborated the importance of such clusters for the whole biofilm formation process in basic cell functions such as replication, transcription, translation and respiration.

**Table 3:** Initial value for concentrations of gene products of the network

Conditions	*y*_0 _= 100*mol*	*y*_0 _= 0*mol*	Graphic Shape
**I**	All Cluster	-	
**II**	Clusters: A, D, F, E, G, H, I & J	Clusters: B, C	

Three set scenarios with different cluster initial concentration 100 or 0 [15]. Scenario I is set to observe the typical cell behavior. Scenario II is set to show network's response time to control biofilm formation. Scenario III is set to see if basic cellular functions are for or against biofilm formation, since the clusters that take part in biofilm formation are off.

We first observed the gene expression dynamics with non-zero initial conditions for all clusters. Interestingly, the global positive regulator FlhDC cluster, capable of regulating the expression of curli and flagella, was found to be repressed after 0.4 s, possibly indicating a negative effect from the FlhD repressors OmpR, RcsCD, hdfR and LrhA. FlhD behavior was also propagated for the H-NS and QseBC positive regulator clusters (Figure 1). Overall, these results suggest that the simultaneous presence of all clusters does not allow biofilms to form owing to repression of key clusters at the onset of the process (clusters A, D and F) [6,18-24]. Consistent with experimental data, cluster F, which is quorum sensing-related, does not follow flagella and H-NS regulator behavior because it is not strongly coupled with OmpR and RcsCD. Phenotypically, these results suggest that quorum sensing requires the strong action of the flagella apparatus in order to play a role during activation; these results were previously shown by González Barrios et al. [4].

**Figure 2:**
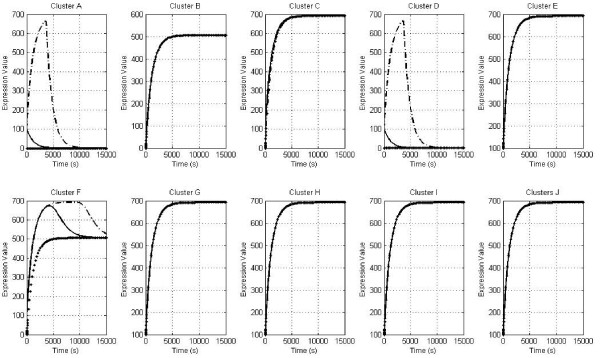
**Profiles for the ten clusters with virtual knockout in cluster B**. Plots for the ten clusters (A-J) show the expression profile (y-axis) vs. time (x-axis) for three scenarios: condition I (-), condition II (-.) and condition 3 (...). Biofilm is not formed under the null scenario because the biofilm positive regulators (clusters A and D) are repressed [expression value (ev) = 0] for three negative regulators (clusters C, G and H) [ev = 700]. However, under scenario II, the biofilm machinery is shown to be activated at 4000s.

*In silico *knockouts were also carried out in order to corroborate the phenotypes previously reported for critical genes during the different stages of the biofilm formation process. First, we wanted to evaluate the effect of deleting the EnvZ/OmpR regulator (cluster B) in order to establish a correspondence between this regulator and the master flagella regulon and determine the potential formation of biofilm when the cluster was knocked out. Overall, we again found no response for biofilm formation since the presence of RcsCD, hdfR and LrhA, which are also reported to be negative regulators in this context, possibly causing the absence of the expression of cluster A, the master flagella regulon (FlhDC), the H-NS regulator (cluster D) and the quorum sensing system (Cluster F). Regarding the initial conditions for this knockout, we noticed that even though the system displays no positive response *in silico *when none is present under the initial conditions, FlhDC almost reaches a positive value, possibly indicating that the initial absence of cluster B could lead the cells to establish biofilm depending on the time response; in other words, the lag time for RcsBC to reach the active state when cluster B is forced to remain shut down. This suggests synergism among the three repressor systems in order to avoid the formation of the biofilm (Figure 2). Moreover, we also found the same results when cluster C (RcsBC) was deleted (data not shown), corroborating this hypothesis of synergism.

Two additional knockouts were analyzed with the aim of identifying the expected positive response from the system. First, we shut down the repressor clusters EnvZ/OmpR and RcsBC, as this has been previously reported to inhibit the flagella response. In this case the master flagella regulon, FlhDC, demonstrated that the biofilm formation is activated and these responses are rapid when the stable state is reached (additional file 1). Also, the major role of the RcsCD and OmpR clusters in regulation was corroborated [6] as these genes remained inactivated, in contrast to hdfR and LrhA. Nevertheless, the fact that A, B, C, D and F present a zero initial condition leads to inactivation of H-NS and QseBC because there is insufficient of the activator FlhCD and a negative phenotypical response (no biofilm formed). Therefore, the biofilm regulation network demands positive action from the inductor clusters or genes directly involved in the positive response such as FlhDC, and the cooperative interaction of the negative regulators [6]. In order to corroborate this conclusion, we obtained the profiles for the cases in which a virtual knockout in cluster A was made and clusters A, B and C were knocked out, and in both cases no biofilm was formed (results not shown). It is important to highlight that virtual knockout of hdfR and LrhA could not be done because these are congregated in fundamental clusters.

Finally, we modeled the case when, in a community of cells that have already formed a biofilm, a signal that activates the OmpR regulator and then RcsCD suddenly arrives (results not shown). The profile displays a biofilm formed until 7000 s and then OmpR begins to produce enough protein to inhibit biofilm formation, concomitantly with RcdCD at 15000 s. Although OmpR starts at 7000 s, biofilm formation (cluster A, D profiles) does not begin to disappear immediately but around 11500 s. This is because OmpR expression is initially absent, and genes for surface organelles are not affected during the time necessary to raise their protein expression.

## Conclusions

In this work we have built a network model of biofilm formation in *E. coli *K-12, using cluster analysis and Boolean network kinetics, obtaining phenotypes already reported. Throughout the modeling, we have used the most important regulators of biofilm formation (FlhD, H-NS, QseBC, OmpR, RcsCD, hdfR and LrhA) under different initial conditions, and obtained enough evidence to demonstrate that networks can efficiently model a complex gene regulatory system such as the biofilm formation in *E. coli *K-12. As in nature, the model simulated successful biofilm formation when the most significant negative regulators (OmpR and RcsCD) are inactive, and when the positive global regulator FlhDC is active.

## Methods

### Data

The Affymetrix Microarray data used were obtained from the NCBI Gene Expression Omnibus (GEO accession: GSE3905) [25,26]. These data were originally obtained by Domka et al. [12]. The data included four time points: 4, 7, 15 and 24 h for cells presenting a biofilm phenotype and for cells in the planktonic state. For this purpose, they grew the two groups of cells in the same reactor in order to eliminate errors associated with different environment conditions. Each set of data included the level of expression of 7312 genes, and in total 8 sets of data were obtained [12].

### Gene clustering

Whole genome profiling allows cluster analysis to be carried out so the genes and clusters that play important roles at each stage can be elucidated. Clustering of gene expression was performed using MultiExperiment Viewer v4.38 [27]. The K-means/medians clustering support algorithm was used to make 10 clusters for each time point with the expression levels for biofilm and planktonic cells. The number of clusters was calculated using the Figure of Merit encompassed in the software, and Euclidean distance was used as the metric distance. The clustering process was restricted to eighty iterations and twenty K-means/K-medians with a threshold percentage of occurrences in same cluster of 0.8. We compared the level of similarity, confirming that most genes were clustered with the same genes for all time points, in order to obtain ten merged clusters.

### Model description

A network model is useful in applications where the information relevant to the problem to be solved is scant or incomplete, but where data are available. Dynamic modeling gives the relevant information about time responses that the ordinary Boolean approximation cannot provide. We then employ an enhanced Boolean network in our model, which reduces the need for large sets of data for network training and displays the dynamic evolution of the network. In the network model, the rate of expression of a gene *i *is given as [17]:

(1) $$\frac{dy_{i}}{dt}=k_{1i}f_{i}-k_{2i}y_{i}$$

Where the first term quantifies the regulatory effect of other genes and the second term describes the degradation. *k_1i _*and *k_2i _*are the rate constants. The decay rate constant *k_2i _*can be expressed through the protein half-life *t_1/2 _*as:

(2) k2=Ln2t12

The regulatory effect of gene *f_i_*, can be represented as follows:

(3) $$f_{i}=\frac{1}{1+\exp\left(-\sum_{j}^{n}w_{ij}y_{j}-\tau_{i}\right)}$$

Where *τ_i _*is the bias (used as a delay parameter for the reaction), *y_j _*represents the concentrations of gene products of the network, and *w_ij _*is the correlation coefficient matrix, called the weight matrix. This matrix describes how various genes in the model affect each other's expression patterns over a period of time. A strong positive correlation indicates that the genes may be co-expressed and have a value around one, and a strong negative correlation indicates that the genes may inhibit each other's expression and have a value around negative one. Therefore the Boolean nature of this model is conserved. Nevertheless, this approach constitutes an improved version as it involves the dynamics of the process. The weight matrix was obtained from different microarrays aimed at studying gene regulation for the *E. coli *K-12 biofilm formation process [2,4-7,12,28-33]. In order to keep track of the genes that play a major role according to the literature, we first determined the interaction among them over time, localizing them in different clusters. We only considered the most relevant genes because these correlations are taken from literature and their functions are well known.

RNA mass balance allows the gene expression profile to be described in the following manner:

(4) dyidt=k1i1+exp(−∑jnwijyj−τi)−Ln2t12yi i,j=1,...,n

We solved the system of ordinary differential equations with the MATLAB^® ^platform using a fourth order Runge Kutta algorithm, with absolute and relative tolerances of 1.0e-006 and 1.0e-003, respectively. To reduce the dimensionality of the solution space we assumed a single time delay *τ_i _*= *τ *for every regulatory interaction. Kinetic parameters were determined based on Gupta et al. [17] (*τ = *1, *t_1/2 _*= 800s, and *k_1i _*= 0.6 mol/s). Simulations were performed using 2.64 GHz Intel Core 2 Duo Processor T7500, 2 GB RAM. (Average time per analysis was 30 s).

## Competing interests

The authors declare that they have no competing interests.

## Authors' contributions

AAS carried out the gene clustering, performed the model and the statistical analysis, and drafted the manuscript. SRR participated in the design of the study and helped to draft the manuscript. AFGB conceived of the study, and participated in its design and coordination and drafted the manuscript. All authors read and approved the final manuscript.

## Supplementary Material

Additional file 1 **Figure 1S - Profiles for the ten clusters with virtual knockout in clusters B and C**. Plots for the ten clusters (A-J) show the expression profile (y-axis) vs. time (x-axis) for three scenarios: condition I (-), condition II (-.) and condition 3 (...). Biofilm is formed under scenarios I and II because the biofilm positive regulators (clusters A and D) are activated [expression value (ev) = 700] in the absence of two most important negative regulators (clusters B and C) [ev = 500]. However, under scenario III, biofilm is not formed since the most important positive regulators is off [ev = 0]. Click here for file

## References

[B1] Schembri M Kjærgaard K Klemm P Global Gene Expression in *Escherichia coli *Biofilms Mol Microbiol 2003 48 1 253 267 10.1046/j.1365-2958.2003.03432.x 12657059

[B2] Vidal O Prigent-Combaret C Dorel C Lejeune P Brobacher E Amabert P Landini A Complex Regulatory Network Controls Initial Adhesion and Biofilms Formation in *Escherichia coli *via Regulation of the csgD Gene J Bacteriol 2001 183 24 7213 7223 10.1128/JB.183.24.7213-7223.2001 11717281 PMC95571

[B3] Arce F Carlson R Monds J Veeh R Hu F Stewart P Lal R Ehrlich G Avci R Tolerance of dormant and active cells in *Pseudomonas aeruginosa Haemophilus influenzae *Biofilms J Bacteriol 2009 191 8 2512 2520 10.1128/JB.01596-08 19218382 PMC2668387

[B4] Gonzalez A Zuo R Hashimoto Y Yang L Bentley W Wood T Autoinducer 2 Controls biofilms Formation in *Escherichia coli *through a Novel Motility Quorum-Sensing Regulator (MqsR, B3022) J Bacteriol 2006 188 1 305 316 10.1128/JB.188.1.305-316.2006 16352847 PMC1317603

[B5] Ren D Bedzyk L Thomas S Ye R Wood T Gene expression in *Escherichia coli *biofilms Appl Microbiol Biotechnol 2004 64 515 524 10.1007/s00253-003-1517-y 14727089

[B6] Prüß B Besemann C Denton A Wolfe A A Complex Transcription Network Controls the Early Stages of biofilms Development by *Escherichia coli* J Bacteriol 2006 188 11 3731 3739 10.1128/JB.01780-05 16707665 PMC1482888

[B7] Vidal O Longin R Prigent-Combaret C Dorel C Hooreman O Lejeune P Isolation of an *Escherichia coli *K-12 Mutant Strain Able To Form biofilms on Inert Surfaces: Involvement of a New ompR Allele That Increases Curli Expression J Bacteriol 1998 180 9 2442 2449 9573197 10.1128/jb.180.9.2442-2449.1998 PMC107187

[B8] Danese P Pratt L Kolter R Exopolysaccharide Production Is Required for Development of *Escherichia coli *K-12 Biofilms Architecture J Bacteriol 2000 182 12 3593 3596 10.1128/JB.182.12.3593-3596.2000 10852895 PMC101973

[B9] Garía-Contreras R Zhang X Kim Y Wood T Protein Translation and Cell Death: The Role of Rare tRNAs in Biofilm Formation and in Activating Dormant Phage Killer Genes PLoS ONE 2008 3 6 1 15 10.1371/journal.pone.0002394 PMC2408971 18545668

[B10] Ren D Zuo R Gonzalez A Bedzyk L Eldridge G Pasmore M Wood T Differential Gene Expression for Investigation of *Escherichia coli *Biofilms Inhibition by Plant Extract Ursolic Acid Appl Environ Microbiol 2005 71 7 4022 4034 10.1128/AEM.71.7.4022-4034.2005 16000817 PMC1169008

[B11] Beloin C Valle J Latour-Lambert P Faure P Kzreminski M Balestrino D Haagensen J Molin S Prensier G Arbeille B Ghigo J Global impact of mature biofilm lifestyle on *Escherichia coli *K-12 gene expression Mol Microbiol 2004 51 3 659 674 14731270 10.1046/j.1365-2958.2003.03865.x

[B12] Domka J Lee J Bansal T Wood T Temporal gene-expression in *Escherichia coli *K-12 Biofilms Environ Microbiol 2006 2 332 346 10.1111/j.1462-2920.2006.01143.x 17222132

[B13] Baldi P Hatfield G DNA Microarrays and Gene Expression: From Experiments to Data Analysis and Modeling 2002 1 Cambridge: Cambridge University Press 143 162

[B14] Silvescu A Honavar V Temporal boolean network models of genetic networks and their inference from gene expression time series Complex Systems 2001 13 54 70

[B15] Sankar M Osmont KS Rolcik J Gujas B Tarkowska D Strnad M Xenarios I Hardtke CS A qualitative continuous model of cellular auxin and brassinosteroid signaling and their crosstalk Bioinformatics 2011 10 1404 12 10.1093/bioinformatics/btr158 21450717

[B16] Franke R Theis FJ Klamt S From binary to multivalued to continuous models: the lac operon as a case study J of Integr Bioinform 14 2010 2151 10.2390/biecoll-jib-2010-151 21200084

[B17] Gupta R Achenie L A Network Model For Gene Regulation Computers and Chemical Engineering 2007 31 950 961 10.1016/j.compchemeng.2006.08.008

[B18] Stanley N Lazazzera B Environmental signals and regulatory pathways that influence biofilm formation Mol Microbiol 2004 52 4 917 924 10.1111/j.1365-2958.2004.04036.x 15130114

[B19] Jubelin G Vianney A Beloin C Ghigo J Lazzaroni J Lejeune P Dorel C CpxR/OmpR Interplay Regulates Curli Gene Expression in Response to Osmolarity in *Escherichia coli* J Bacteriol 2005 187 6 2038 2049 10.1128/JB.187.6.2038-2049.2005 15743952 PMC1064031

[B20] Wei B Brun-Zinkernagel A Simecka J Prüß B Babitzke P Romeo T Positive regulation of motility and *flhDC *expression by the RNA-binding protein CsrA of *Escherichia coli* Mol Microbiol 2001 40 1 245 256 10.1046/j.1365-2958.2001.02380.x 11298291

[B21] Francez-Charlot A Laugel B Van Gemert A Dubarry N Wiorowski F Castanié-Cornet M Gutierrez C Cam K RcsCDB His-Asp phosphorelay system negatively regulates the *flhDC *operon in *Escherichia coli* Mol Microbiol 2003 49 3 823 832 12864862 10.1046/j.1365-2958.2003.03601.x

[B22] Vianney A Jubelin G Renault S Dorel C Lejeune P Lazzaroni J Escherichia coli tol and rcs genes participate in the complex network affecting curli synthesis Microbiology 2005 151 2487 2497 10.1099/mic.0.27913-0 16000739

[B23] Majdalani N Gottesman S The Rcs Phosphorelay: A Complex Signal Transduction System Annu Rev Microbiol 2005 59 379 405 10.1146/annurev.micro.59.050405.101230 16153174

[B24] Sherlock O Dobrindt U Jensen J Vejborg R Klemm P Glycosylation of the Self-Recognizing *Escherichia coli *Ag43 Autotransporter Protein J Bacteriol 2006 188 5 1798 1807 10.1128/JB.188.5.1798-1807.2006 16484190 PMC1426561

[B25] Imbeaud S Auffray C 'The 39 steps'in gene expression profiling: critical issues and proposed best practices for microarray experiments Drug Discov Today 2005 10 17 1175 1182 10.1016/S1359-6446(05)03565-8 16182210

[B26] Gene Expression Omnibus (GEO accession: GSE3905) http://www.ncbi.nlm.nih.gov/geo/query/acc.cgi?acc=GSE3905

[B27] Saeed A Bhagabati N Braisted J Liang W Sharov V Howe E Li J Thiagarajan M White J Quackenbush J TM4 microarray software suite Methods in Enzymology 2006 411 134 193 16939790 10.1016/S0076-6879(06)11009-5

[B28] Beloin C Ghigo J Finding Gene-Expression Patterns In Bacterial Biofilms Trends Microbiol 2005 13 1 16 19 10.1016/j.tim.2004.11.008 15639627

[B29] Pratt A Kolter R Genetic analysis of *Escherichia coli *biofilms formation: roles of flagella, motility, chemotaxis and type I pili Mol Microbiol 1998 30 2 285 293 10.1046/j.1365-2958.1998.01061.x 9791174

[B30] Herzberg M Kaye I Peti W Wood T YdgG (TqsA) Controls Biofilm Formation in *Escherichia coli *K-12 through Autoinducer 2 Transport J Bacteriol 2006 188 2 587 598 10.1128/JB.188.2.587-598.2006 16385049 PMC1347309

[B31] Delisa M Wu C Wang L Valdes J Bentley W DNA Microarray-Based Identification of Genes Controlled by Autoinducer 2-Stimulated Quorum Sensing in *Escherichia coli* J Bacteriol 2001 183 18 5239 5247 10.1128/JB.183.18.5239-5247.2001 11514505 PMC95404

[B32] Francez-Charlot A Castanié-Cornet M Gutierrez C Cam K Osmotic Regulation of the *Escherichia coli *bdm (Biofilm-Dependent Modulation) Gene by the RcsCDB His-Asp Phosphorelay J Bacteriol 2005 187 11 3873 3877 10.1128/JB.187.11.3873-3877.2005 15901715 PMC1112044

[B33] Hagiwara D Sugiura M Oshima T Mori H Aiba H Yamashino T Mizuno T Genome-Wide Analyses Revealing a Signaling Network of the RcsC-YojN-RcsB Phosphorelay System in *Escherichia coli* J Bacteriol 2003 185 19 5735 5746 10.1128/JB.185.19.5735-5746.2003 13129944 PMC193970

