# Supplementary figures and images for: A network model for biofilm development in Escherichia coli K-12
Source: Theor Biol Med Model. 2011 Sep 22;8:34. doi: 10.1186/1742-4682-8-34 (PMC3224578; doi:10.1186/1742-4682-8-34)

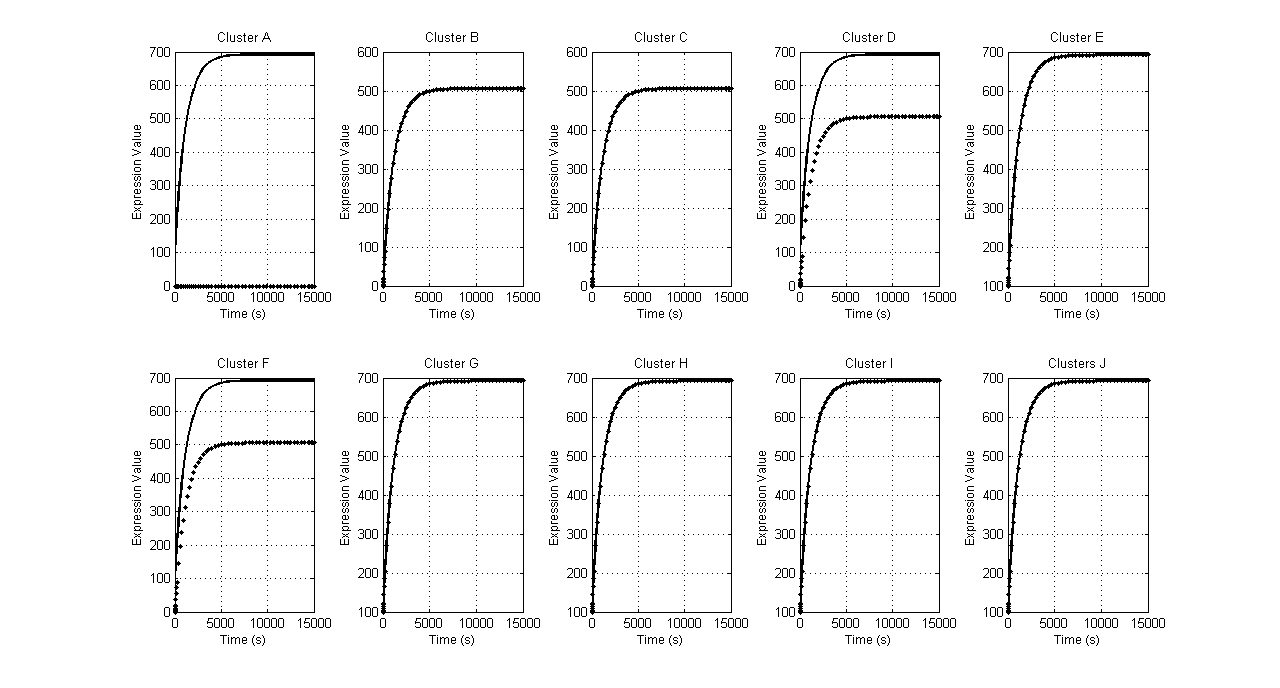

Supplement: Additional file 1 — Figure 1S - Profiles for the ten clusters with virtual knockout in clusters B and C. Plots for the ten clusters (A-J) show the expression profile (y-axis) vs. time (x-axis) for three scenarios: condition I (-), condition II (-.) and condition 3 (...). Biofilm is formed under scenarios I and II because the biofilm positive regulators (clusters A and D) are activated [expression value (ev) = 700] in the absence of two most important negative regulators (clusters B and C) [ev = 500]. However, under scenario III, biofilm is not formed since the most important positive regulators is off [ev = 0]. [file 1742-4682-8-34-S1.PNG]
